# Trustworthy artificial intelligence and ethical design: public perceptions of trustworthiness of an AI-based decision-support tool in the context of intrapartum care

**DOI:** 10.1186/s12910-023-00917-w

**Published:** 2023-06-20

**Authors:** Rachel Dlugatch, Antoniya Georgieva, Angeliki Kerasidou

**Affiliations:** 1 Ethox Centre, Nuffield Department of Population Health, University of Oxford, Old Road Campus, Headington, Oxford, OX3 7LF UK; 2 Nuffield Department of Women’s & Reproductive Health, University of Oxford, Level 3, Women’s Centre, John Radcliffe Hospital, Oxford, OX3 9DU UK

**Keywords:** Trust, Trustworthiness, Artificial intelligence, Ethical design, Public perception, Cardiotocography

## Abstract

**Background:**

Despite the recognition that developing artificial intelligence (AI) that is trustworthy is necessary for public acceptability and the successful implementation of AI in healthcare contexts, perspectives from key stakeholders are often absent from discourse on the ethical design, development, and deployment of AI. This study explores the perspectives of birth parents and mothers on the introduction of AI-based cardiotocography (CTG) in the context of intrapartum care, focusing on issues pertaining to trust and trustworthiness.

**Methods:**

Seventeen semi-structured interviews were conducted with birth parents and mothers based on a speculative case study. Interviewees were based in England and were pregnant and/or had given birth in the last two years. Thematic analysis was used to analyze transcribed interviews with the use of NVivo. Major recurring themes acted as the basis for identifying the values most important to this population group for evaluating the trustworthiness of AI.

**Results:**

Three themes pertaining to the perceived trustworthiness of AI emerged from interviews: (1) trustworthy AI-developing institutions, (2) trustworthy data from which AI is built, and (3) trustworthy decisions made with the assistance of AI. We found that birth parents and mothers trusted public institutions over private companies to develop AI, that they evaluated the trustworthiness of data by how representative it is of all population groups, and that they perceived trustworthy decisions as being mediated by humans even when supported by AI.

**Conclusions:**

The ethical values that underscore birth parents and mothers’ perceptions of trustworthy AI include fairness and reliability, as well as practices like patient-centered care, the promotion of publicly funded healthcare, holistic care, and personalized medicine. Ultimately, these are also the ethical values that people want to protect in the healthcare system. Therefore, trustworthy AI is best understood not as a list of design features but in relation to how it undermines or promotes the ethical values that matter most to its end users. An ethical commitment to these values when creating AI in healthcare contexts opens up new challenges and possibilities for the design and deployment of AI.

**Supplementary Information:**

The online version contains supplementary material available at 10.1186/s12910-023-00917-w.

## Introduction

As artificial intelligence (AI) is becoming increasingly common in the medical and healthcare landscape, more importance is being placed on developing AI that is ethical and trustworthy. Despite consensus that trustworthiness is essential for the successful design, application, and acceptability of AI [1, 2], what constitutes trustworthiness in relation to AI, and who should determine and define it, is less evident.

Approaches to technology design that integrate people’s values into it, such as value-sensitive design [3], embedding values [4], embedded ethics [5, 6], ethics by design [7], and Responsible Research and Innovation [8], ostensibly place people at the forefront of the design process. Despite this emphasis on human values, however, perspectives of the public and direct stakeholders (i.e., those who interact directly with the technology in development) are often absent from the research and discourse on ethical AI design [9, 10]. Meanwhile, international organizations and regulatory bodies are attempting to foster public confidence by publishing guidelines and codes of ethics; however, these initiatives are aimed at facilitating public trust rather than designing AI that is *worthy* of the public’s trust [11].

This research investigates the views of direct stakeholders to explore the meaning and role of trust in the design, development, and application of AI in a medical context. By artificial intelligence, we refer to data-driven and computer-based systems and software able to perform tasks normally associated with intelligent beings. Using a speculative case study, this paper sheds light on the perspectives of birth parents and mothers^1^ regarding the introduction of AI-based cardiotocography (CTG) in the context of intrapartum care. Based on data collected in seventeen interviews with birth parents and mothers who were pregnant and/or had given birth in the last two years, this research places users’ perspectives at the forefront of the discourse on the ethical and trustworthy design and application of AI.

### Trustworthy AI

Trustworthiness is a characteristic of an agent that denotes the agent’s competence to perform an action and her moral attitude or commitment towards those who depend on her to perform said action [13, 14]. Being trustworthy is something more than just being predictable and reliable; rather, it signifies a moral characteristic or virtue [13]. For this reason, some have argued that trustworthiness is an inappropriate characteristic to attribute to inanimate objects, such as AI systems, as one cannot ascribe moral attitudes or virtues to agents that are not themselves moral agents [15–17]. Being reliable, namely acting in a predictable and consistent way, is much more fitting for agents that can act but do not qualify as moral agents. Others have defended the notion of trustworthy AI by arguing that attributing trustworthiness only to human agents reveals a narrow definition of trust [18], ignores academic and disciplinary disagreement regarding the conceptualization of trust and the role of non-human agents [19], and fails to take into account the common use of the words ‘trust’ and ‘trustworthy’ [19].

Whilst we agree with the argument that trustworthiness is relevant only to moral agents, we also acknowledge that in everyday language one might refer to an object or a technology as being trustworthy, as an (indirect) reference to the belief that the people and processes involved in making and deploying that object or technology are themselves trustworthy. It seems that it is in this more indirect understanding that the term ‘trustworthy AI’ is used in documents such as the guidelines published by European Commission’s High-level Expert Group on Artificial Intelligence [1]. The seven key requirements articulated in the document refer to standards and principles that need to be incorporated in the ways in which AI systems are developed and deployed, including structures for governance and oversight. Furthermore, in the White Paper published in 2020 by the European Commission entitled *On Artificial Intelligence: A European Approach to Excellence and Trust*, these key requirements are referred as the foundation upon which an ‘ecosystem of trust’ should be based, which is critical for ‘giv[ing] citizens the confidence to take up AI applications and give companies and public organizations the legal certainty to innovate using AI’ [2].

It is the acknowledgement that trust and trustworthiness can be an important enabling factor when it comes to the acceptability and use of AI by the public [2, 20–23] that has driven the proliferation of published principles and guidelines for ethical AI by public bodies, private companies, and research institutions [11]. A study conducted by Jobin et al. [24] revealed 84 such documents. Yet, as the authors conclude, this plethora of guidelines highlights the need for a global agenda for ethical AI, which respects cultural and moral pluralism, but also the necessity of developing clear implementation processes.

Following from this short analysis, one way of understanding the notion of trustworthy AI is as the designing, developing, and implementing of AI tools and models in ways that adhere to and reflect certain values and principles conducive to warranting trust. In so far as the values embedded into AI tools are those valued by end-users, one could argue that building them in would indicate trustworthiness. This understanding is predicated on the view discussed in the next section that values, including ethical values, can be reflected in the way in which technologies are developed and used.

### Ethical values in design

One way of implementing ethical values and principles into technology is by bringing ethical considerations, including stakeholders’ perspectives regarding ethical implications of new technologies, right from the start of the design process and throughout the lifecycle of technology development and deployment. This attitude reflects a generally accepted, albeit not uncontroversial view [25], that technologies are not value-neutral, but rather, through their development and deployment, technologies can promote or undermine certain values [26, 27].

A number of methodologies have been proposed to facilitate the incorporation of ethics and ethical values into the design of data-driven technologies, such as AI. Value sensitive design [3], embedding values [4], embedded ethics [5, 6], ethics by design [7], and Responsible Research and Innovation [8], are some of these methodologies that argue for the importance of integrating ethics into the entire process of AI systems development and deployment, as a way of ensuring these systems and technologies are ethical and socially responsible, and also able to respond to value change. However, even when ethics is incorporated into the design processes of AI systems, the voices of important stakeholders, such as end-users, might still be excluded thus making these tools, as well as the ethical recommendations regarding their development and deployment, less relevant to the people on the ground [28]. As such, relevant stakeholders, e.g. main user groups, should act as a primary source for identifying values to be promoted within technology design and deployment.

### The context for the study: artificial intelligence and cardiotocography interpretation

Cardiotocography (CTG) is used by obstetricians and midwives to monitor the wellbeing of the fetus during pregnancy and/or during labor, especially for high-risk pregnancies. Ultrasound traducers are placed on the birthing parent’s or mother’s abdomen by a clinician, enabling the CTG to continuously record the fetal heart rate and uterine contractions [29]. The purpose of this electronic fetal monitoring is to identify babies who may suffer from hypoxic injury (lack of oxygen), and to assist clinicians in identifying whether an intervention—such as a caesarean section or assisted vaginal birth (with the use of ventouse and/or forceps)—is necessary [30]. The idea is that timely, corrective decisions can be made by healthcare professionals during labor if they can assess fetal wellbeing and the individual risk of hypoxia more reliably [31].

Although CTG was introduced into clinical practice in the late 1960s and has been a mainstay in obstetrics since then, there is little to no evidence that CTG has been effective in improving perinatal outcomes [32]. For example, rates of infant mortality and cerebral palsy (a complication associated with hypoxic-ischemic encephalopathy) have not been reduced with the use of CTG [30]. Meanwhile, rates of cesarean sections have risen [30, 33]. Despite being endorsed for usage by major professional organizations, some studies seem to link widespread CTG usage with increased cases of mortality and morbidity to both birth parents and babies in high-income countries [33, 34]. Use of CTG has also been criticized for restricting movement [30], being uncomfortable or not allowing women to stay in the bathtub, which has over time pushed the main manufacturers [35, 36] to develop wireless and water-proof solutions.

One reason that CTG evaluations are unreliable is that the readings themselves are highly subjective [37]. Experts have been shown to interpret the same CTG trace differently from one another and even to contradict themselves [38]. Despite the introduction of guidelines to improve this ‘high inter- and intra-observer variability,’ however, CTG evaluation is still highly inconsistent [39]. Moreover, these guidelines are based on patterns of fetal heart rate that are imprecise, static, and otherwise insufficient for reliable CTG interpretation [37].

Due to the limitations of using CTGs to prevent adverse labor outcomes, there has been a growing interest in developing improved ways for CTG interpretation and beyond [37]. Artificial intelligence and machine learning approaches, for example, are being proposed as alternatives to conventional CTG interpretations, aiming to improve clinical practice through more accurate and objective assessments [40].

Leading in this domain, a computer-based analysis method under development is the Oxford System (OxSys), based on the CTG and labor data from nearly 100,000 prior births. OxSys incorporates known clinical risk factors (demographics and comorbidities) jointly with the automated analysis of the CTG and is ‘trained’ to estimate the risk for severe compromise at birth (fetal or neonatal death, neonatal encephalopathy, hypoxic-ischemic encephalopathy and the need for resuscitation and neonatal intensive care). The aim is to assist clinicians in detecting more reliably those at risk and communicating this with the birthing parent and their family, facilitating joint decision making to prevent severe perinatal outcomes. However, OxSys aims to also minimize the false positive rates and reduce as much as possible the rate of unnecessary interventions with their inherent risks [41].

In the context of OxSys and this particular research, the general term AI is used, but more specifically, OxSys is a data-driven computer-based algorithm/software, trained and tested with the data of hundreds of thousands prior births. The size of the data is crucial because adverse outcomes are rare and many clinicians will see in their lifetime only a few such cases. But OxSys allows advanced algorithms to ‘learn’ from the data of such rare events as well as that of many healthy births. After learning/training, the information from the prior data is distilled into the memory of the computer and, when a new birthing parent presents in front of the healthcare professional, their individual CTG trace and clinical risk factors together are analyzed by OxSys. The tool then provides an objective, data-driven estimate for the risk for this individual baby based on how it compares to the data of the large population in OxSys’s ‘memory.’ Essentially, OxSys is one more tool in the hands of clinicians which they can use together with the birthing parent and their specific clinical context to discuss the pros and cons of clinical options for intervention and make a joint decision based both on their individual needs and the data-driven objective risk estimate.

With their lives and their babies’ lives at stake, birth parents and mothers have the most to gain and the most to lose with the introduction of new technologies in perinatal care. In addition, given that there are unique challenges in labor decision making—such as making pressurized decisions while the laboring parent is in pain or on drugs—and also that expectations and preferences may differ from one person to the next, it is important to consider the wide spectrum of individual needs and perspectives to develop, design, and implement trustworthy AI. However, patient views and experiences are distinctly lacking from research on CTG monitoring [42]. Our team has sought to integrate the parents’ views and experiences within the core of OxSys’s development, leading to this study.

## Methods

### The study

This study is part of an NIHR i4i Product Development Award that is developing an independently validated, trustworthy, and clinically relevant AI-based CTG (OxSys 3.0).

This study investigated the perspectives of birth parents and mothers regarding the introduction of AI-based CTG during term labor, with a focus on the issue of trust. Semi-structured interviews were conducted with seventeen birth parents and mothers who were pregnant and/or had given birth in the last two years, and who were based in England. The aim of these interviews was to capture what direct stakeholders believe the ethical issues are in the use of AI, the meaning and role of trust in the development and use of AI-based CTG, and how it should be used in practice.

The decision to conduct qualitative research, which allowed birth parents and mothers to delineate the ethical issues pertaining to trust, had a twofold purpose. First, it served to amplify users’ voices in the literature on the trustworthy development and deployment of AI in healthcare. Given that female participation in medical research pales in comparison to their male counterparts [43, 44] and that research that falls under the umbrella ‘women’s health’ is often neglected and marginalized in the first place [45–47], enabling minoritized voices to generate and contribute to knowledge production might go some way also to address an ‘epistemic injustice’ [48]. Additionally, focusing on the views of people most affected by technological interventions in healthcare sheds new light on themes of significance beyond obstetrics and which may be extrapolated to other areas of medicine and the development of trustworthy AI more broadly.

Second, this qualitative approach facilitated the understanding—and therefore, paved the way for the incorporation—of users’ values into the design and implementation of an actual AI decision-support CTG software, OxSys 3.0. This research is part of a larger project that aims to improve clinical decision-making around labor management and CTG monitoring. Although any value-based design should be an iterative process, this study acted as the initial stepping stone in that process for the development and deployment of OxSys. It also represents a modest step in a broader movement to develop trustworthy and ethical AI in a society witnessing increasing technological interventions.

### Participants

Seventeen birth parents and mothers were recruited for semi-structured interviews. The criteria for participation was that interviewees were based in England and were pregnant and/or had given birth in the last two years. Participants were recruited in the following ways: known contacts in National Childbirth Trust (NCT) groups, distribution of research flyers via email and social media (Twitter, Facebook groups and pages), cold contacting pregnancy and new parent support groups, posting research flyers in local community centers, and snowballing. Descriptions of the research given to prospective participants via email and social media stated that no knowledge of artificial intelligence was necessary and that interviews would be primarily based on personal experience and hypothetical situations. This point was made to avoid attracting only prospective participants who were already well-versed in the subject of artificial intelligence or comfortable talking about new technology. Furthermore, although most recruitment was done online because of the Covid-19 pandemic and because participation was open to people based anywhere in England, the recruitment strategy also specifically targeted areas of Oxford with high socioeconomic and ethnic diversity (e.g. East Oxford). 10 of the 83 neighborhood areas of oxford are among the 20% most deprived areas in England [49]. We distributed flyers to community centers and open spaces that serve this particular population of Oxford. Additionally, flyers stated that the research was being conducted by the University of Oxford and that participants would receive £20 vouchers. The flyer was submitted to MS IDREC alongside all the other project materials. It was reviewed and approved by MS IDREC.

We also endeavored to make participation inclusive by reaching out to support groups for parents from systemically disadvantaged backgrounds (i.e., due to ethnicity and/or socioeconomic status). Of these seventeen participants, six were pregnant at the time of interviewing, three of whom were pregnant for the first time. Out of the eleven participants who were not pregnant, nine had birthed one child, and two had given birth more than once.

### Interviews

Because this study was conducted during the Covid-19 pandemic, for health and safety reasons, recruitment and interviews took place online, on Microsoft Teams. The researcher (RD) allowed participants to schedule interviews to suit their needs. Interviews often took place around lunchtime, when participants’ children were down for a nap or when their partners were able to look after the children. Some interviews were conducted while the interviewee was nursing. Most calls were taken with video enabled, except for a few participants who opted to turn their cameras off, usually due to poor internet connection. All calls were audio recorded on an external encrypted device, an Olympus DS-9000. Interviews lasted 43 min on average.

Prospective interviewees were given Participant Information sheets and Informed Consent Forms to read before interviews took place. Informed consent was then taken verbally at the beginning of the call by the researcher (RD), who read through the Informed Consent Form aloud. Participants were also given permission to have a support person or partner on the call due to the potential sensitive nature of the conversation, but only two participants chose to do so.

AI was not used in interviewees’ care. As such, interviews focused on participants’ lived experiences of pregnancy and childbirth, as well speculative scenarios involving AI-based CTG in intrapartum care. Interviewees were first asked to reflect on their relationships with healthcare professionals, how decisions were made about their care, and their feelings about any encounters with technology (including but not limited to CTG). Then, participants were asked to share what came to mind when hearing the phrase ‘artificial intelligence,’ how they imagined AI could be used in medical contexts, and their feelings toward it. This was to assess participants’ level of knowledge pertaining to AI and any preconceptions they may have without first providing them with a definition which could influence their responses.

In the final portion of the interview, participants were given a brief description of an AI-based CTG (OxSys). Because participants were not experts in AI or CTG, it was necessary to describe OxSys in laymen’s terms rather than being overly technical. What was important to communicate to participants was that (i) an intelligent data-driven and computer-based fetal monitoring software would provide a risk assessment, and (ii) that the risk assessment would not necessarily determine the course of action taken but act as another piece of information for both the healthcare professional and laboring person to consider. The initial description was deliberately kept vague so as not to influence interviewees’ responses and see what issues participants raised on their own. For example, where the data was sourced from and what data points were included were left out of this initial description to see if participants were concerned about this issue without first being prompted. The same is true for who was developing OxSys, whether the OxSys considered other risk factors apart from heartrate, whether it has been proven to be more accurate than healthcare professionals, and more. These, amongst other issues, were later introduced by the interviewer (RD) if participants did not raise them unprompted.

After discussing how this type of technology differed or matched with their own ideas about AI, as well as their initial impressions and concerns, participants were then asked a series of questions to probe what they consider to be the ethical issues of introducing such a system, as well as what characteristics would make it (*un*)trustworthy. Participants were encouraged to reflect on how introducing this kind of AI-based decision-support tool might impact the care they receive, their relationships with their doctors and midwives, their own decision-making capabilities, and their overall experience of giving birth. Given that the AI in question was not used in their care, this final portion of the interview was speculative. Nevertheless, it was contextualized and grounded in their lived experiences of pregnancy and childbirth. The interview guide for the semi-structured interviews is included in this publication as a additional file. Note that because of the semi-structured nature of the interviews, the interview guide was used to provide a general direction to the interview. During the interview, participants talked about what they felt was important, which the researcher then followed up. This means that each interview was different from the next, although all of them covered the general areas included in the interview guide.

While attention was paid to issues pertaining to trust and trustworthiness, semi-structured interviews allowed for flexibility and empowered participants to define the issues of greatest importance to them. Many participants said that they enjoyed having an opportunity to share their experiences, including the moments of pregnancy and childbirth that were a source of anger, frustration, and/or sadness, because they had never been given the opportunity to do so.

### Analysis

All interviews were transcribed verbatim by a transcription service that has a signed confidentiality agreement with the University.The transcription service received audio recordings from the researcher (RD) and returned written transcripts via an encrypted data exchange program.. RD then uploaded these transcripts to NVivo, a data analysis software, to organize and manage interview transcripts. Analysis was performed using thematic analysis [50], a method for systematically identifying and interpreting thematic patterns in qualitative data sets. After RD and AK read and familiarized themselves with the interview transcripts, RD coded the transcripts. Coding is a term in thematic analysis that refers to assigning a label to parts of interview text that speak to a particular topic or concept. Then, RD reviewed these codes and grouped them by overarching theme and then again into sub-themes. RD and AK met regularly to review these themes, to define and redefine them, and also to discuss how they related to and informed our research questions. RD and AK agreed on the themes most relevant to the question of what constitutes trustworthy AI in the context of intrapartum care. The themes selected for this paper were the ones most reinforced and echoed by our participants and were also most relevant to the project/work package designed by AK and the overall research project designed by AG.

Participants have been given pseudonyms for this paper. Direct quotations have been edited for readability only.

## Results

The findings of this study are split into three sections: (1) trustworthy AI-developing institutions, (2) trustworthy data from which AI is built, (3) trustworthy decisions made with the assistance of AI. These three themes emerged from interviews as being important to the evaluation of the trustworthiness of AI.

### Trustworthy AI-developing institutions: the importance of promoting public good

The trustworthiness of the institution developing AI was perceived by participants as relevant to the overall trustworthiness of the AI system itself. When discussing the importance of who develops AI for use in medical contexts, participants emphasized the need for AI being developed and deployed for public good. Public good was expressed as medical tools and technologies that will benefit the birth parent/mother and baby and are equally available to all. Tools developed with the aim of generating profit were viewed as antithetical to promoting public good. Almost unilaterally, participants associated university researchers with developing technologies for public benefit, and private companies as being mainly motivated by profit. For this reason, patients characterized trustworthy development as that which had been carried about by independent and/or university researchers instead of for-profit private companies.

Below is a response from Rosie, a first-time pregnant woman, to a question about whether who had developed the AI in question would matter:

…you want to know that it’s not just someone trying to make money. You’d want it to be something independent, where you are like, ‘Is this people who are trying to make money, or is this people who are trying to make better outcomes for women in labor?’ That would definitely mean something.

As Rosie’s comment highlights, being motivated by financial gains was juxtaposed with maximizing better outcomes for birth parents and mothers. The idea that these two goals are incompatible was reiterated by most interviewees. Additionally, participants tended to believe that university researchers were more invested in improving health outcomes than those in private companies, such as Google. Lily, mother to several children, made these institutional associations explicit:

Yeah, I think if it was a university, that’s more reassuring than just Google. I just wonder what Google’s best interest would be though. Would it be financial? Whereas university researchers like yourself, you are actually quite interested in the health of the mother or whatever, whereas I don’t know if Google would be.

Jennifer, mother of one, reiterated these associations by expressing her distrust of monetization, something she does not affiliate with university-based research:

Whereas when it’s come from a university backing, I think I trust much more that the data’s been harvested in a way that’s not always had in the back of its mind, how do we monetize that?

As these comments reveal, the perceived difference between universities and private companies was not seen only as a signifier of a pragmatic difference between these institutions (e.g., with respect to their institutional aims and priorities), but was viewed as a ‘moral’ difference. In other words, participants perceived universities as being more *trustworthy*, in virtue of pursuing public good, than private companies. Moreover, this perceived difference impacted the way in which participants perceived the overall trustworthiness of the AI system itself. AI developed by trustworthy institutions (e.g., universities) was perceived as more trustworthy than AI developed by untrustworthy institutions (e.g., private companies).

Despite this conviction that the trustworthiness of the AI-developing institution matters, however, many interviewees conceded that if the AI system was already at the point of use in the NHS, it would indicate that it had been proven to improve outcomes for birth parents and mothers. In other words, participants believed that AI implemented in the NHS would have already been proven to satisfy their ethical requirement of promoting public good. Consider the following comments:

You kind of trust in your hospital that they would only get something that has been made to help kind of thing, and [made] by people we trust. –Rosie

But if it got to the point that it was in healthcare on a sort of everyday basis, I would probably trust that it had got to that place [of improving outcomes], maybe naively, I don’t know. –Mary

That participants expressed trust in their healthcare system to prioritize their wellbeing is not a surprise, given that public trust in the NHS, particularly in relation to managing patient data, remains high [51]. What these quotes do reveal, however, is that there is more than one level at which public trust operates and in which commitment to public good can be demonstrated (or undermined): first, at the level of the institutions seeking to develop AI tools, and second, at the level of public institutions tasked with assessing and implement technologies on the ground. Given this linkage between the perceived trustworthiness of institutions and the perceived trustworthiness AI they develop and/or implement, this finding has potential implications for the role of institutions in the introduction of AI in healthcare.

### Trustworthy data from which AI is built: reliable, unbiased, and consistent

Although necessary, an institutional commitment to public good was not sufficient for interviewees to deem the AI system that the institution developed as trustworthy. Participants acknowledged that while *who* builds AI is significant, AI can only be only as good as the data it is built *from*. As such, trustworthy AI needs to be derived from ‘trustworthy data,’ a phrase used by one participant but implied by many participants. ‘Trustworthy data’ in these discussions means reliable data, or data that is unbiased and inclusive.

Several participants spoke about their concerns regarding biases in datasets used for developing and training AI systems, thus outlining what would constitute *un*trustworthy data, and by extension, *un*trustworthy AI. More specifically, there was a recognition amongst interviewees that certain population groups, especially Black and Minority Ethnic (BAME) birth parents and mothers, suffer worse maternal outcomes. Although not their only concern, participants worried that data might not be as inclusive of BAME populations and therefore any systems developed using this data would be unreliable for them. As a result, the outputs of the AI would be less accurate for these groups, and thus they would not consider the AI itself as trustworthy. What is interesting is that interviewees associated AI trustworthiness with data reliability in terms of accuracy and inclusivity, even when they themselves did not belong to these marginalized groups.

The following quote establishes that participants want to know that data itself is trustworthy:

I think that’s another thing, that people want to know that data is trustworthy. —Jennifer

Building on this idea, another interviewee expressed trustworthy data in terms of its reliability:

              Obviously I’d want to know, where’s this data from? Is it reliable? –Rosie.

Although participants spoke of reliability in relation to data, further discussions elucidated that data itself was not participants’ main concern in and of itself. Reliability of input (data) was inextricably linked with reliability of output (in the case of AI-based CTG, a risk assessment), even amongst participants who openly acknowledged their limited knowledge of AI. As the following quote illustrates, reliability of output was framed in relation to bias, with unbiased outcomes stated as the ultimate goal to be achieved:

This is my completely limited knowledge, so this could be rubbish, but my understanding is AI is only as good as its inputs […] I think it would have to be based on a wide breadth of inputs and experiences for it to have proper and unbiased outcomes. —Mary

One person added that even though the perceived moral character and intentions of the institution developing the AI was of moral significance, good intentions cannot guarantee reliability, particularly in relation to the breadth of data used. Consider the following comment:

I would trust the ethics of a university doing it more, but again, fundamentally, if the issue is the data that you put in, then the university’s data can’t actually be any better than the private company’s. I do generally trust independent research more than private research. But I don’t think that it removes all of the issues. –Taylor

When probed further about reliability and data, Taylor brought up recent reports on BAME maternal health outcomes and mortality rates [52–54]. Like Taylor, several participants had read these reports in the news and raised the issue of implications of biased data sets for marginalized populations. Moreover, interviewees expressed the importance of having inclusive datasets so as not to reproduce these social and health inequalities. Underlying this need for reliability, then, is a desire for equity and solidarity in healthcare, in the form of ensuring the same reliability of outcome across all population groups. Taylor makes the point as follows:

I have some of the same concerns that I was talking about already around how do you make sure it’s not a biased data set? ... Just having done the research in passing, it comes up on stuff about maternal outcomes for Black and ethnic minority women and the fact that data will be in the system in the way it is currently. So there’s not a way round that kind of thing. [Minority groups] have a lot of complex medical stuff going on all at the same time [and] what the world looks like for those people is often really quite a long way from what the mainstream world looks like. And making decisions that are good for the majority of people often doesn’t work for people who are already in a significantly marginalized position. —Taylor

Another participant expressed a similar sentiment, adding that even though this would not affect her personally as a white woman, she nevertheless has concerns for others:

So deaths within mothers of Black and ethnic minorities far outstrip those of white individuals, and nobody knows why [….] And there was a big push to try and understand why those individuals are experiencing such a different end result or, you know, labor. And no one can really sort of pinpoint why that is. So I would have concerns, not for myself, but for others that those unknown factors aren’t being taken into consideration because it’s the unknowns when you’re trying to ask a computer to spit out the answer but you don’t know what the input is. So that’s another dynamic. I think there’s a point in there. –Fran

Finally, despite these concerns about biased data sets and unreliable outcomes for BAME parents, participants nevertheless shared an optimism that assessments performed by AI could be more reliable than those performed by humans. As the following comments show, in this context, reliability was framed in terms of consistency of outcome—namely, that the same inputs will generate the same outputs.

I think I would feel that [the] use of a large body of data by software could be really, really powerful and it could eliminate some of that personal bias stuff that everybody has. You know, healthcare professional scientists have it as well. –Emma

To move it to being more evidence based, rather than it just being one person’s opinion, sounds fantastic to me. Obviously, I had quite a negative experience of every day coming away with a different feeling based on whichever individual I was talking to. So, to know that it’s actually just based on data, rather than one person’s opinion. That’s, that’s really reassuring. –Jennifer

As such, despite their concerns about the data AI is taking as inputs, participants generally felt positively about AI’s capacity for consistency of outputs. More importantly, however, reliability of outcomes for all population groups was perceived as important for the overall trustworthiness of the AI system. Therefore, while generating consistent outputs is one piece of this puzzle, the data from which AI is built must also fairly represent all population groups and be trustworthy in and of itself for the AI built from it to be considered trustworthy.

### Trustworthy decisions made with the assistance of AI: human mediation and limits to machine autonomy

Even though participants acknowledged that AI could generate more consistent outputs, they nevertheless expressed concerns about the capacity of AI to make holistic assessments. For this reason, alongside the recognition that healthcare professionals have relevant clinical experience, participants asserted that AI should act as a supplement to, rather than a replacement for, healthcare professionals’ clinical judgments. According to the research sample, trustworthy decisions made with AI require human input and oversight, as it is humans (i.e., healthcare professionals) who are able to fully engage with the human (i.e., patient) in front of them. Furthermore, participants articulated a strong preference for decisions to be mediated by a healthcare professional for their ability to personalize care, including incorporating patients’ values into the decision-making process and communicating directly with patients.

Several participants articulated this point about AI supplementing healthcare professionals’ clinical decision making:

I think possibly I would always see it as a complement or a supplement to a healthcare professional that I trusted, not necessarily a replacement. –Jennifer

I think as long as it’s well researched and I suppose as long as there’s a human eye to cast over it a little bit so you’re not purely at the sole mercy of a machine, as long as there’s some little bit of human contact there, then I think I would have a lot of confidence in it. –Chelsea

Additionally, when participants were asked whether they would trust their healthcare professional or AI in a case of clinical discrepancy, participants overwhelmingly said that they would trust the healthcare professional’s decision over AI. Several interviewees highlighted the importance of making holistic decisions, something they believed humans were more capable of than AI; others cited clinical experience of healthcare professionals as a relevant factor, too. The following quotes are interviewee responses to the question of whether they would trust their healthcare professional or AI to make a final decision about their care (such as needing to intervene in labor):

My gut would say the healthcare professional because they’re seeing me in the here and there, like now. They’ve got my notes. They can overlay that with what might have happened before. They know what’s happened in the run-up to that that day. –Fran

I think I’d trust the doctor more. Because they have experience and they’re skilled. […] Because they can see, they can actually *see*. And also, a lot of emotions in our bodies can be interpreted in the wrong way by the machine. Maybe you’re just nervous or anxious and [AI-based CTG is] recording distress. –Tiffany

The desire and preference for personalized care, understood as care that took into consideration patients’ needs, values, and preferences as individual persons, was something that our participants returned to again and again. In discussing personal experiences of being treated more like a statistic rather than an individual person even in the current system, they expressed their concerns that the introduction of AI might exacerbate such attitudes. Consider this quote explaining how a decision for induction was made and communicated to one of our participants, Taylor:

I’m booked in for an induction in a week’s time that I don’t agree with. I know that the NICE guideline says that if there isn’t a specific set of problems, that the induction should be in about Week 39. I know that. I work in mental health myself. I’m pretty scientifically literate. And I’ve said to the registrars every time, ‘Look, this is what I want to happen. I’m happy with increased monitoring from Week 37. Can we do it this way?’ The registrars have said, ‘That sounds completely reasonable,’ on every occasion. They’ve taken it up to the consultant, who has never seen or spoken to me, and he’s said no. […] So, fundamentally, I think it’s because it’s hospital policy. […] I just want to feel like I got to make the decisions and that I understood why they were getting made and that they felt like they were made in my best interests and my baby’s best interests. –Taylor

Although this experience does not involve the use of AI, Taylor still felt that this ‘algorithmic decision’—in this case, in the form of hospital policies and guidelines—could detract from the type of holistic and personalized care that birth parents and mothers consider appropriate and ultimately trustworthy. By introducing AI into the care system and then relying on it to make final decisions for patient care, our participants’ feared that they would end up losing even more opportunities for holistic and personalized care, as there would be no healthcare professional with whom they would be able to discuss and reason. Although AI might be able to make certain care decisions for patients, it offers very little opportunity to communicate back, and being able to communicate their views and be confident that they are taken into account when planning their care was something participants in this study valued.

Finally, empathy was revealed as a final consideration for why participants would want decisions to be made with a doctor. Consider the following comments:

I think it’s just all about that care and connection really, you want to feel like you’re being well looked after and a machine doesn’t always give you that sort of feeling 100%; you still want someone there to, kind of, physically hold your hand a little bit. –Chelsea

… [W]e need technology, but of course like that type of assistance will always be limited in many regards, and I think when you are an expecting mother you always need that kind of human touch. You want to be recognized and seen as a human. So I think like the healthcare professionals play an important role in reassuring mothers, and fathers, and explaining [things to] them … is just really, really important. It helps a long way when you are in a stressful and a non-stressful situation. So those things are very important no matter what technology will be introduced and how it is used. It’s just very important that there’s a good, good kind of communication going on at all times. –Charlie

Ultimately, the consensus amongst participants was that people should have the final say in any decision-making process. Nevertheless, this point does not suggest a weariness about healthcare professionals incorporating AI in their decision-making process. What is does suggest is that trustworthiness of AI is not independent from the way in which it is implemented on the ground but reliant on it.

## Discussion

This paper has explored the ways in which birth parents and mothers conceptualize trustworthy AI in the context of intrapartum care, filling a gap in the research on patient perspectives. A small number of qualitative studies have been published to date on patient perspectives regarding the introduction of AI tools in various parts of healthcare [55–57] but none, to our knowledge, on the specific area of intrapartum care that is examining the issue of trust.

Promotion of public good, reliability, fairness, personalized and holistic care, human mediation, and empathy were all deemed as necessary to the perceived trustworthiness of AI. 

These findings shed new light on themes pertaining to the trustworthiness of AI and raise questions about how human values can be interpreted into the trustworthy development, design, and implementation of AI. Our findings chime with those of a large five-country survey based study regarding trust in AI [58] and shed new light on the topic by providing a more in-depth and nuanced perspective of these issues. They also raise questions about how human values can be interpreted into the trustworthy development, design, and implementation of AI. The remainder of this section explores some of these possibilities and the ethical challenges that may arise in the value-sensitive design process.

### Institutions and public good

Patients and birth parents interviewed for this study expressed the view that public institutions such as universities and the NHS are more trustworthy than private companies because of their commitment to promoting public good, whereas private companies are driven by profit maximization. They attributed a moral significance in the motivation and aims of these stakeholders, which they viewed as part of their moral character and perceived trustworthiness. This position is not unique to participants of this research; other studies have also demonstrated the public’s skepticism regarding private companies’ motivations in the context of health [59]. Although there is philosophical disagreement about whether collective actors such as institutions and companies are moral actors and therefore warrant trust (or distrust) [60], this reported attitude towards universities and the NHS chimes with theoretical approaches to public trust as the trust warranted towards public institutions that aim at providing some kind of public good or benefit [61]. Motivation to serve a public good can be understood as an indication of an institution’s moral motivation and character, and thus indicate trustworthiness even if it cannot, in itself, guarantee trust.

However, research and development of medical products and devices, including medical AI, can be costly. Even if original research is led by universities using national healthcare system data, bringing these products to the patients at scale often requires budgets and expertise found in the private sector. This raises an ethical challenge for public institutions about how to involve and collaborate with private companies for the development and deployment of medical technologies, including AI, whilst preserving their trustworthiness and promoting public good.

It is important to bear in mind that public trust operates on multiple levels. As participants revealed, although they perceive public research institutions such as universities to be more trustworthy, they also trust the NHS to introduce only those technologies that are effective on the ground and beneficial to patients. This is because they rely on its processes for checking and validating the efficiency and effectiveness of new technologies, but also on its solidaristic character for making decisions that benefit all patients [62].

Collaborations between public and private institutions when it comes to the introduction of AI in healthcare could be perceived as trustworthy not because the private company would develop an interest in public good, but because a trustworthy institution would be overseeing the technology and its implementation on the ground, such as NHS Trusts or regulatory bodies that check and approve the introduction of new technologies (e.g. MHRA). A number of studies have tried to articulate criteria that would make such partnerships trustworthy. Horn and Kerasidou (2020) maintain that norms such as commitment to public good should be incorporated into these agreements. They suggest that requirements such as preferential access to technologies developed using NHS patient data, limiting the use of patient data to for-public-benefit purposes, and transparency and effective resolutions of conflicts of interests as well as use of trusted research environments to manage data use outside the NHS could promote trustworthy collaborations. Graham (2021) points at transparency, accountability, representation, and ensuring social purpose even if this at times might come to the expense of commercial gains, as ways of ensuring trust and confidence in public-private collaborations that aim at producing data-driven medical technologies like AI [63].

### Data, reliability, and fairness

In the literature on CTG interpretation, reliability is expressed as one of the main potential benefits of incorporating machine learning and AI [40]. In this context, reliability refers to consistency (i.e., the same inputs producing the same outputs, unlike with humans who are subjective in their CTG interpretation), as well as accuracy (i.e., more thorough and nuanced data/inputs to generate more precise outputs). A more reliable CTG would detect more adverse perinatal outcomes while also minimizing false positive rates and unnecessary interventions [41].

Reliability, in terms of accuracy and consistency of output, was important to participants of this study. However, they also framed reliability primarily in relation to the concept of bias , thus linking reliability with the values of equity and fairness in the over- or under-representation of certain populations in datasets as well as output. In their view, a biased dataset, particularly one that did not include data from marginalized populations, such as BAME parents, was perceived as contrary to reliability. According to participants, in order for AI tools to be reliable, data used in their development should account for the heterogeneity of relevant patient populations so as not to reproduce existing social inequalities, such as worse maternal outcomes for BAME patients. With (health) equality being seen as essential for reliability, rather than a separate and additional value, the measurability of reliability is linked to justice, not consistency and accuracy alone. Furthermore, participants of this study pointed at another value, that of solidarity, which is less often mentioned in discussions regarding reliable AI [64, 65]. By framing reliable AI as something that preserves and promotes mutual support and equal access to benefits for all populations, participants in this study might be reflecting the solidaristic character of their national healthcare system [62], as well as more widespread conceptions of healthcare as a form of public good to which all should have access [66, 67].

There has been a considerable attention to issue of bias in the development and deployment of AI, including medical AI, and how to best understand, interpret and incorporate values such as fairness and equality in AI systems [68–71]. To address these issues, any weaknesses and bias in the dataset should be identified so its limitations can be understood. Then, developers can identify ways of overcoming these limitations. This might include continuous collection of more robust and inclusive data, so that the dataset itself is more representative of all population groups. Furthermore, there may be an argument for introducing fairness and equity type of considerations at the stage of product approval for medical use. One requirement might be that AI tools should produce and declare confidence or reliability scores for the different population groups to which these tools would be relevant. This way, those assessing (e.g. MHRA, FDA)—and later on, those using the tools (e.g., healthcare professionals)—might be in better position to make decisions regarding the reliability and effectiveness of these new technologies on different patient groups. Of course, this solution raises its own set of questions that relate to the way that principles of justice, fairness, and solidarity should be understood and translated into practice. For example, if AI is only reliable for some groups, should it not be used on others? If it benefits white birth parents and mothers only, then the tool could exacerbate health outcome disparities. Should there a reliability threshold AI must pass on all relevant population groups in order to be implemented on the ground?

Although this research does not provide solutions to these challenges, it nevertheless provokes the ethical questions that need to be addressed.

### Decisions and holistic, patient-centered care

Participants were unanimous in their preference for decisions regarding their care being made by humans, not autonomous machines. This point reiterates the need for decisions to be mediated by healthcare professionals, but fundamentally reveals participant perceptions regarding the limits of AI in considering them as persons rather than a collection of data, as well as delivering patient-centered care [72]. There are a few ways in which design features of AI might be able to address this desire for personalized care. For example, the ability to input individual risk factors is a potential design decision that could address clinical relevancy of assessments and recommendations. Additionally, while the ability to input and personalize clinical risk factors is the most obvious way that AI-based CTG can increase personalization through design, it should also be noted that patients may have values, preferences, and risk thresholds that differ from their healthcare professionals. There is an argument to be made, then, that AI also requires value flexibility [73] to improve personalization capacities, not just flexibility in clinical inputs alone. In the case of AI-based CTG, this feature might look like adjusting for a patient’s risk threshold before an intervention is suggested.

However, although AI might be able to improve upon some aspects of personalization, the healthcare professional remains essential making individual assessments and providing patient-centered care. As Taylor’s experience highlighted, if personalization is primarily grounded in data derived from population groups (e.g., diabetics, birth parents and mothers over a certain age, etc.), patients may still feel like they are being reduced to data points and not being treated individually. There is also an argument that patient preferences and values should be considered and incorporated separately from AI-based clinical assessments. In addition, patients also want the ability to communicate back with their healthcare professionals and be part of a shared decision-making process, which requires dialogue, communication, and empathy. Ultimately, then, it is important not to focus on AI design alone but how its implementation on the ground can also enable healthcare professionals to perform more personalized, holistic, empathetic, and patient-centered medicine.

Participants’ understanding of AI as a tool at a healthcare professional’s disposal, at least in the case of AI-based CTG, aligns with its intended use case: as a decision-*support* tool, rather than as an autonomous decision *maker*. If AI is meant to be supportive rather than a replacement for healthcare professionals, guidelines should be put in place so that healthcare professionals do not over-rely on AI. Moreover, if healthcare professionals are meant to integrate this AI into their care, more research is needed on how clinical decisions are made so that this AI system can be seamlessly integrated into their practice to improve upon decision-making processes, including shared decision making with patients.

AI is often seen as a solution to inefficiency. It is thought of as something that can relieve time pressures on clinicians and alleviate the burden of staffing shortages. It is even imagined that as AI becomes more prominent in healthcare, the relationship between healthcare professionals and patients will become less important. However, despite the ever increasing kinds of technologies used in healthcare, it is evident that the trusting relationship between healthcare professionals and patients is still relevant. One participant even said that they would always see AI as a medical supplement ‘to a HCP I trusted.’ As such, developing that trusting relationship between patient and clinician is still of utmost importance—and potentially of greater significance—when introducing new technology into the healthcare space.

For this reason, any time saved with AI should be reinvested in the healthcare professional-patient relationship. This is not only so there is time and space for empathy and human-centered care, but also for improved (more holistic and personalized) clinical decision making.

### Implications for OxSys Development

This research is part of a larger project to develop an AI-based CTG, OxSys. The results of this qualitative study were presented to the whole OxSys research team and discussed during group meetings. A workshop was also organized with members of the ethics group, patient involvement group, and development group to discuss the findings in more depth and consider further steps and take-home messages. During these meetings a number of action points were identified and discussed. In light of the here presented findings, the team discussed, firstly, potential strategies regarding the financing of the future development of OxSys and considered what kind of partnerships the project team should pursue. Secondly, the bias, fairness, and reliability points raised by the study participants led to a discussion regarding how the model can be continuously reevaluated and whether the model can correct for bias in the data. Furthermore, the possibility of including a confidence score for different populations as an add-on to the model was considered. Finally, and in relation to the third finding presented, the OxSys team was encouraged to see that their aim to build a decision-aid tool rather one that could be used to replace clinical expertise chimed with the views of our participants. A fruitful discussion evolved from this point regarding how risk scores could be presented to facilitate decision-making. For example, we discussed whether to present risk scores using a traffic-light color scheme, how to allow for personalization of risk assessments, whether it is possible to codify personal risk-perceptions regarding certain interventions (e.g. use of ventouse, forceps or caesarian section), and how to build in a functionality to allow users to focus on specific parameters of the risk score analysis. A number of points for actions to help refine the planned feasibility study were also extrapolated from our findings, including the point of appropriate training of healthcare professionals in using the tool.

### Limitations

Although we found that we had reached data saturation after a preliminary thematic analysis of these seventeen interviews, we nevertheless recognize the limited size of our study. Further research with a greater sample size would make it easier to extrapolate themes and arguments about what birth parents and mothers (and patient groups more broadly) consider ethical and trustworthy AI. Additionally, this study did not collect personal data about participants, such as ethnicity, age, educational attainment, sexuality, and socioeconomic background. While abstaining from this kind of data collection protects participants’ privacy, it nevertheless limits the capacity for intersectional analysis. Future research may benefit from collecting this kind of data and exploring the ways in which people’s identities and experiences inform their values and world views, including where they might converge and diverge by population group (and within them, too).

Prospective participants were told that there was no need to have expertise in AI to participate in our research. However, it is worth acknowledging that research participants are a self-selecting group and may therefore be more interested in discussing the topic of our research, as well as open to talking to university researchers. Nevertheless, many interviewees explicitly stated that they felt uninformed about AI and even silly sharing what first came to mind when they thought of it (one participant laughed at herself when answering, ‘little robots on wheels’); yet, even these participants were able to think through some of ethical and practical issues that may arise with introducing new technology into maternity care. It is not possible to ascertain from our data whether our participant group were more informed or educated on issues that pertain to AI than the general population, and we concede that further research could explore the relationship between educational attainment and perceptions of AI, as well as birthing experiences more broadly. Moreover, although people who choose to participate in university research may be biased in favor of public institutions, the finding that the people find public institutions more trustworthy than private institutions, especially in a healthcare context, is corroborated by other research [59].

Finally, this research was premised on a speculative design scenario rather than lived experiences with the AI in development. In part, this was due to a practical limiting factor, namely that at the time of interviews, OxSys was not being trialed with birth parents and mothers. However, speculative research is also an important step in the process of ethical design; it enables values and perspectives of users to inform early iterations of the AI undergoing development, rather than being reduced to an afterthought. This type of approach has been referred to as ‘proactive orientation toward influencing design’ [3]. Nevertheless, further research that investigates people’s lived experiences with AI is also needed. Creating and implementing trustworthy AI is an iterative process that requires an understanding of the ethical challenges at every stage of its development [10]; therefore, the collection and evaluation of users’ perspectives should be sustained and carried forward in future research.

## Conclusion

This paper explored birth parents’ and mothers’ perspectives regarding the trustworthiness of AI-based CTG, and more broadly, patient perspectives on the ethical issues associated with introducing AI in healthcare. The topics that participants of this study felt were most relevant to the theme of trustworthiness were institutions, including which institutions develop and implement AI; data, particularly the dataset from which AI is developed and how representative it is of various population groups; and AI-assisted decision-making, especially regarding the relative role(s) and autonomy of healthcare professionals and machines in the process. However, even though the participants of this study spoke about AI and their concerns regarding the introduction of new technology in intrapartum care, what they were really talking about was their concerns about the provision of healthcare as a whole. Underscoring these conversations about AI were the values that birth parents and mothers want to preserve in healthcare more broadly: solidarity, equality, fairness, and reliability, as well as values and practices like the promotion of public good, patient-centered care, holistic care, and personalized medicine in the healthcare system.

Ultimately, to ask what constitutes trustworthy AI in intrapartum is to consider what constitutes trustworthy healthcare in the first place. The way in which the development, design, and implementation of AI threaten or bolster the values that matter most to its users will determine how these key stakeholders evaluate its trustworthiness. Paradoxically, this means that the trustworthiness and ultimate success of AI is not dependent on its technology alone but on *people*—the user groups who are positioned to delineate the ethical values to preserve and maximize, and the developers, policy makers, and healthcare professionals who translate these values into design and practice.

## Electronic supplementary material

Below is the link to the electronic supplementary material.

Additional File 1: Interview Topic Guide for Pregnant women/women who have given birth in the past 24 Months

## Data Availability

All relevant data are within the manuscript. The interview guide is included as a supplementary file. The data generated and analyzed during the current study are not publicly available as participants did not consent to data archiving and data will be destroyed 3 years after the end of the study as per MS IDREC requirements. Specific data is available from the first author on request.

## Footnotes

1 Following the clinical and language guidelines by Brighton and Sussex University Hospitals Trust [12], we refer to this patient group as ‘birth parents and mothers.’ This approach of using ‘gender-additive language’ means that gender neutral language can coexist with language of womanhood and motherhood. This language allows transgender and non-binary parents to be included in perinatal discourse while not erasing the experiences of women. Not only is using gender-inclusive language morally responsible, but it also best represents the identities of participants of this study. At least one participant identified as non-binary, and as such, ‘birth parents and mothers’ is a more representative descriptor of the participants of this study than ‘mothers’ or ‘women.’

## List of abbreviations

AI: Artificial intelligence
BAME: Black, Asian, and minority ethnic
CTG: Cardiotocography
MHRA: Medicines and Healthcare products Regulatory Agency
FDA: Food and Drug Administration
