# Supplementary material for: Trustworthy artificial intelligence and ethical design: public perceptions of trustworthiness of an AI-based decision-support tool in the context of intrapartum care
Source: BMC Med Ethics. 2023 Jun 20;24:42. doi: 10.1186/s12910-023-00917-w (PMC10283298; doi:10.1186/s12910-023-00917-w)
Supplement: Supplementary file 1 — Additional File 1: Interview Topic Guide for Pregnant women/women who have given birth in the past 24 Months [file 12910_2023_917_MOESM1_ESM.pdf]

INTERVIEW TOPIC GUIDE FOR PREGNANT WOMEN/WOMEN WHO HAVE GIVEN BIRTH IN THE PAST 24 MONTHS

Notes

This is a topic guide. Specific wording of questions will be adapted as the project progresses and according to the participants' answers. This topic guide will be used as a map for the researchers to ensure that they cover all important topics, even if they will have to adapt either the order of the questions or the way they ask the question.

Introduction

- Researcher/s introduce the aim of the research
- Researcher asks if participant has any further questions regarding the project and their participation
- Researcher confirms consent
- Research reminds participant that they are free to refuse to answer any questions, or to stop the interview at any stage
- Researcher thanks participant for taking time to do this interview

Questions

Decision-making

Is this your first pregnancy?

How long is it since you gave birth to your last baby?

How did you find the experience of pregnancy and giving birth?

How much contact did you have with healthcare professionals (midwifes, consultants)?

How did you find these interactions? [probe: reassuring, stressing?] Did you feel supported during your pregnancy by your healthcare team?

Were there any particular decisions you had to make about your pregnancy/delivery?

How did you feel when you were making these decisions? [probe: confident, stressed, the burden of the decision…?]

Tell me about the doctors/midwives involvement in these decisions? How did you feel about their involvement? [probe: did they guide you more/less, happy/not happy with conversations, felt in safe hands]

During labour and the delivery of the baby, do you know/remember if your doctors used any specific technologies to monitor you and/or the baby? Did you ever think you'd like to know more about these tools? Yes/No/Why?

[Transition: As you might have heard, nowadays more and more artificial intelligence tools are being developed to be used in healthcare, including in the care of women in labour. So, for example, researchers are developing a tool very similar to the one often used during labour to monitor the baby's heartrate so healthcare professionals can make a decision about whether they need to intervene and get the baby out quickly. The main difference is that the AI tool would be using data from many previous deliveries to guide the doctor's decision.]

Artificial intelligence

So, how would you feel if your doctor was using such a tool when they were looking after you and your baby? Why?

What might be your thoughts about your doctor using AI to care for you? Why? [probe: for example, would you feel happy with their decision to intervene, if that decision was made based on the AI tool?]

What might be your concerns? Why? [probe: for example, would you have any concerns about their expertise, or that about the amount of time they spend looking after you?]

Would knowing who is developing this tool, e.g. if it is university researchers or an international private company like Google or IBM, make any difference to how you might think about it? Why?
